# Density and Viscosity of CO_2_-Loaded Aqueous 2-Amino-2-methyl-1-propanol (AMP) and Piperazine (PZ) Mixtures

**DOI:** 10.1021/acs.jced.4c00403

**Published:** 2024-11-08

**Authors:** Diego Morlando, Ardi Hartono, Hanna K. Knuutila

**Affiliations:** Department of Chemical Engineering, Norwegian University of Science and Technology, Trondheim N-7491, Norway

## Abstract

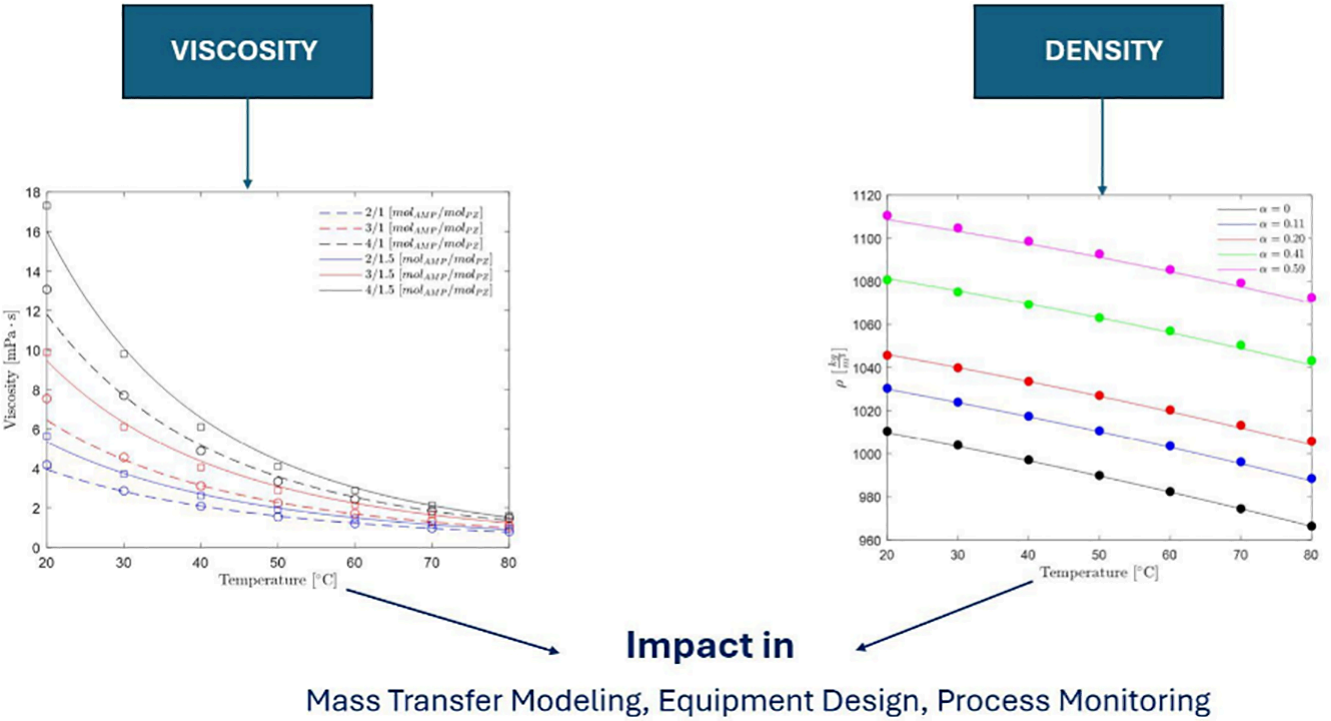

Densities and viscosities of aqueous 2-amino-2-methyl-1-propanol (AMP)/piperazine (PZ) solutions with and without CO_2_ are measured from 20 to 80 °C at ambient pressure. Redlich–Kister-based correlations are proposed for the excess molar volumes and viscosity deviation of the binary and ternary mixtures. Empirical correlations are developed to quantitatively describe the effect of CO_2_ on the density and viscosity of the aqueous AMP/PZ solutions. The experimental data and correlations developed can be used in the design and simulation of AMP/PZ-based CO_2_ capture absorption plants.

## Introduction

Chemical absorption using amine-based solvents is the most mature technology for post-combustion carbon capture. A blend of 3 mol/dm^3^ 2-amino-2-methyl-1-propanol (AMP) and 1.5 mol/dm^3^ piperazine (PZ), also known as the CESAR1 solvent, has been proposed as a new benchmark for this technology.^1^ In our previous work, we carried out a literature review to identify the current experimental gaps in the CESAR1 system.^2^ We outlined that density and viscosity data for CO_2_-loaded and CO_2_-unloaded aqueous AMP/PZ solutions at the CESAR1 concentration constitute an important experimental gap. Viscosity and density data are essential to building process models that can be used in the design of CO_2_ capture plants. Viscosity affects the mass transfer between the gas phase and the liquid phase and therefore impacts directly the absorber performance. Furthermore, these physical properties are needed in the design and optimization of the heat exchangers in the process.

Fu et al.,^3^ Dash et al.,^4^ Murshid et al.,^5^ Paul and Mandal,^6^ Samanta and Bandyopadhyay,^7^ and Sun et al.^8^ measured the viscosity for CO_2_-unloaded AMP/PZ aqueous solutions. Fu et al.^3^ only measured viscosity for CO_2_-loaded AMP/PZ aqueous solutions up to 50 °C; however, viscosity and density data at the CESAR1 concentration currently miss in the open literature.

**Figure 1:**
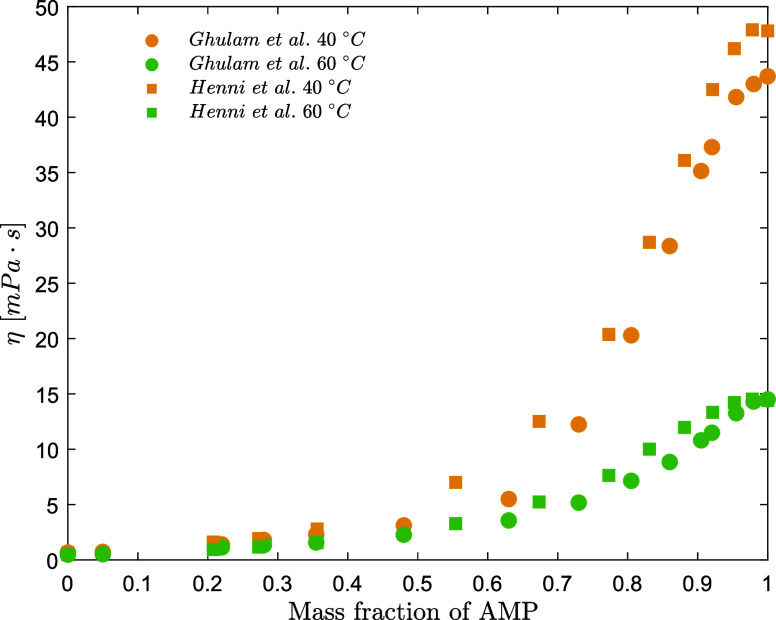
Viscosity literature data for aqueous AMP solutions: solid circle, Ghulam et al.;^10^ solid square, Henni et al.^9^

Some experimental data for density and viscosity of CO_2_-loaded and CO_2_-unloaded aqueous AMP, PZ, and AMP/PZ solutions are available in the open literature, but high disagreement has been found among the data sets available. Murshid et al.^5^ and Fu et al.^3^ measured the viscosity of aqueous CO_2_-unloaded AMP/PZ at 20 mass % AMP and 10 mass % PZ, and Paul and Mandal^6^ measured the viscosity of aqueous CO_2_-unloaded AMP/PZ at 21 mass % AMP and 9 mass % PZ. The experimental results are not in good agreement with each other, and therefore, extrapolation at higher concentrations, such as at the CESAR1 concentration, is difficult. Additionally, large discrepancies in the viscosity data for aqueous AMP solutions have been detected. The concentration of the AMP solvent may change in a real operation due to different factors, such as solvent evaporation and degradation. It is therefore important to develop flexible physical property models accounting for amine concentration changes. Henni et al.^9^ and Ghulam et al.^10^ measured the viscosity of aqueous AMP solutions in a concentration range from 0 to 100 mass % and up to 70 and 60 °C, respectively. These two datasets at high AMP concentration deviate significantly from each other as shown in Figure 1. The deviation between the two datasets is estimated to be up to 20%.

This work provides viscosity and density data for aqueous AMP/PZ solutions as a function of temperature, AMP/PZ molar ratio, and CO_2_ concentration. Additionally, some viscosity and density experiments for aqueous AMP solutions are performed. Models based on Redlich–Kister (RK) equations have been developed to describe the effect of amine concentration and temperature on the physical properties of CO_2_-unloaded solutions.^11^ The data have also been fitted to correlations to quantitatively describe the effect of CO_2_ on the above-mentioned properties. Finally, physical property models for aqueous AMP and aqueous PZ solutions are developed since they are needed to model the ternary (AMP-PZ-H_2_O) and quaternary systems (AMP-PZ-H_2_O-CO_2_).

## Experimental Section

In the open literature, the CESAR1 solution is defined on a molar basis,^12^ and therefore, the design of the experiments and all the concentrations are reported in the molarity scale (mol/dm^3^) and as mass fraction.

### Chemicals

Aqueous solutions of AMP and AMP*/*PZ were prepared by dissolving the amines with deionized water on a Mettler Toledo MS6002S with an accuracy of 1 × 10^–5^ kg. Solutions, defined on a molar basis, were prepared using a 1dm^3^ flask, stirred overnight, and then used for experiments. The chemicals used in this work, their providers, and their purity are reported in Table 1. Deionized water from the university-centralized system was used without further purification. The conductivity of deionized water was measured during the experimental campaign and found to be 1.6 μS/cm.

**Table 1:**
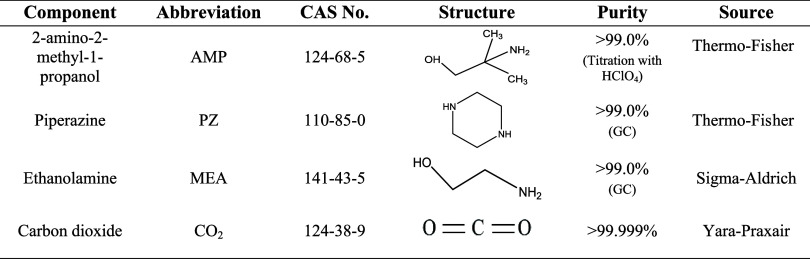
Chemicals Used in This Work^a^

a The purity was obtained by the COA (Certificate of Analysis).

**Table 2:**
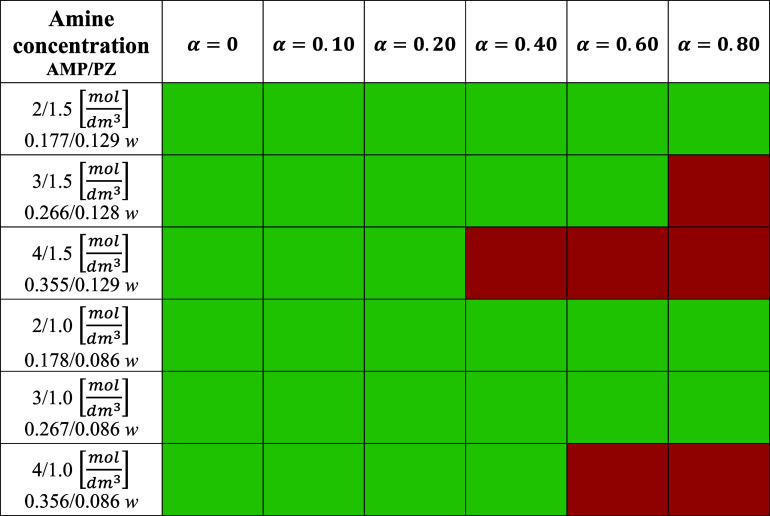
Prepared CO_2_-Loaded Aqueous AMP/PZ Solutions^a^

a The green color indicates no precipitation. The red color indicates that precipitation occurred during solvent preparation.

CO_2_-loaded solutions were prepared gravimetrically by bubbling CO_2_ into the aqueous amine solutions under ambient conditions. A Mettler Toledo MS6002S with an accuracy of 10^–5^ kg was used to evaluate the weight change due to the addition of CO_2_. The CO_2_ loading was then determined by inorganic carbon analysis (IC). The sample was diluted with Milli-Q water, acidified with H_3_PO_4_, and then sparged with synthetic air. A nondispersive infrared sensor (NDIR) was used to measure the concentration of CO_2_. The uncertainty of the analytical value was quantified using a standard aqueous solution of sodium hydrogen carbonate (NaHCO_3_).

Acid–base titration was used to quantify the amine content. In the tables, the concentrations and loadings are based on analytically determined values. Based on the analytical results of the standard prepared for the IC, the CO_2_ loading, α, defined as , is estimated to have a standard relative uncertainty *u*_r_(α) = 0.02.

**Table 3:** Datasets Used in the Fitting for the Models of the Density of Aqueous AMP, PZ, and AMP-PZ Solutions

reference	amine concentration [*w*]	temperature [°C]
AMP–H_2_O		
this work	0.05–0.95	20–80
Na et al.^18^	0–1	20–60
Henni et al.^9^	0–1	25–70
Chan et al.^19^	0–1	25–80
PZ–H_2_O		
Murshid et al.^5^	0.017–0.10	20–60
Derks et al.^23^	0.054–0.146	20–60
Sun et al.^8^	0.020–0.08.0	30–40
AMP–PZ–H_2_O and AMP–PZ–H_2_O–CO_2_		
this work	[AMP] = 2–4 mol/dm^3^	20–80
	*w*_AMP_ = 0.177–0.356	
	[PZ] = 1–1.5 mol/dm^3^	
	*w*_PZ_ = 0.086–0.129	

**Table 4:** Datasets Used in the Fitting for the Models of the Viscosity of Aqueous AMP, PZ, and AMP-PZ Solutions

reference	amine concentration [mass %]	temperature [°C]
AMP–H_2_O		
this work	5.0–95	20–80
Henni et al.^9^	0–100	25–70
PZ–H_2_O		
Muhammad et al.^28^	1.7–10.3	30–60
Samanta and Bandyopadhyay^7^	1.7–10.3	20–60
Derks et al.^23^	5.4–14.6	20–60
Sun et al.^8^	2.0–8.0	30–40
AMP–PZ–H_2_O and AMP–PZ–H_2_O–CO_2_		
this work	[AMP] = 2–4 mol/dm^3^	20–80
	*w*_AMP_ = 0.177–0.356	
	[PZ] = 1–1.5 mol/dm^3^	
	*w*_PZ_ = 0.086–0.129	

**Figure 2:**
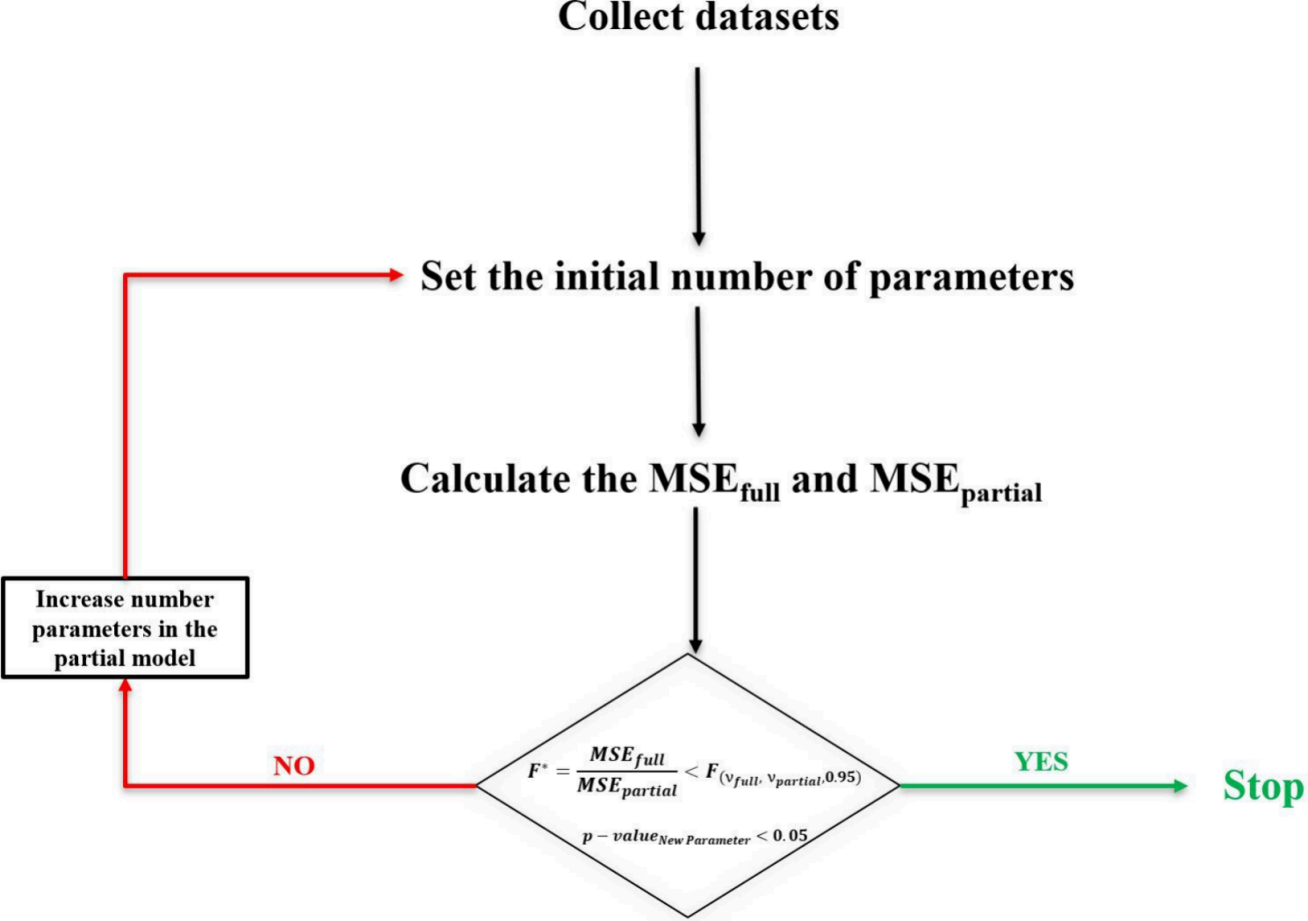
Algorithm for the parameter optimization of the developed models.

### Equipment Validation and Methodology

A DMA 4500 density meter coupled to a Lovis 2000ME viscosity meter by Anton Paar was used in this work to perform the viscosity and density measurements. The built-in data analysis by the manufacturers was used to treat the data. The DMA 4500 was calibrated at 20 °C by air and ultrapure H_2_O supplied by the vendor. The Lovis 2000ME was calibrated using ultrapure H_2_O. The viscometer is based on a rolling ball principle. A capillary was filled with a gold-coated stainless-steel ball to cover the viscosity measurements from 0.2 to 65 mPa·s. The procedure for the experiments includes an air check (i.e., measuring air density), water measurements, sample measurements, and in the end, new water measurement. This is to check any possible drifting during the measurements of the unknown samples and to provide additional cleaning before a new experimental run. For experiments with CO_2_-loaded solutions, selected samples were analyzed before and after experiments at high temperature, 60 °C, to ensure that the CO_2_ loading did not change during the experiments. The maximum deviation of the loading was 1.4%, which is lower than the calculated experimental uncertainty for the CO_2_ loading. Additionally, a visual camera in a DMA 4500 was used to detect the possible stripping of CO_2_ and its consequent bubble formation. Finally, the apparatus was validated by measuring viscosity and density data for 30 mass % aqueous ethanolamine (MEA). The absolute average relative errors (AARDs) for the density measurements are 0.03 and 0.05% for the Hartono et al.^13^ and Han et al.^14^ datasets, respectively. The AARDs for the viscosity measurements are 1.7 and 1.4% for the Hartono et al.^13^ and Arachchige et al.^15^ datasets, respectively. Hartono et al.^13^ reported the maximum repeatability of their measurements to be ± 2%, and therefore, the methodology was considered validated. Additional information regarding the validation is available in Figures S1–S3 and Table S1.

The standard uncertainty of the density measurements, *u*(ρ), was estimated to be 0.4 kg/m^3^. Information on the uncertainty analysis can be found in the Supporting Information. Valtz et al.^16^ measured the viscosity of aqueous amine solution using a Lovis 2000ME. They reported an expanded relative deviation, *U*_*r*_(η) = 0.01, as suggested in the Lovis 200ME manual. In this work, the standard relative uncertainty for the viscosity measurements, *u*_r_(η), was estimated to be 0.05 based on the validation results obtained with MEA, which is the most used reference system for aqueous amine solution for CO_2_ capture. The standard temperature uncertainty, *u*(*T*), and standard pressure uncertainty, *u*(*P*), are, respectively, 0.01 K and 0.3 kPa. The standard uncertainty for the amine mass fraction was calculated, and more information is available in the Supporting Information.

**Table 5:** Density Data of Unloaded AMP Solutions in Water at Atmospheric Pressure^a^

	ρ [kg/m^3^]						
*T* [°C]	*w*_AMP_ = 0.05	*w*_AMP_ = 0.15	*w*_AMP_ = 0.30	*w*_AMP_ = 0.50	*w*_AMP_ = 0.70	*w*_AMP_ = 0.90	*w*_AMP_ = 0.95
20	997.7	997.8	999.7	995.7	981.9	951.7	942.6
30	995.0	994.3	994.2	988.0	972.9	943.5	934.4
40	991.4	990.0	988.2	980.2	965.7	935.2	926.1
50	987.1	985.0	981.7	972.5	957.2	927.0	917.8
60	982.1	979.4	974.8	964.4	947.1	918.4	909.4
70	976.5	973.3	967.9	956.3	937.9	909.7	900.3
80	967.9	966.6	960.3	947.5	928.6	900.5	891.5

a Standard uncertainties are *u*(*P*) = 0.3 kPa, *u*(*T*) = 0.01 K, and *u*(ρ) = 0.4 kg/m^3^, and expanded uncertainties are *U*(ρ) = 0.8 kg/m^3^, with a 0.95 level of confidence (*k* ∼ 2), and *u*(*w*_AMP_) = 0.0001.

**Table 6:** Viscosity Data of Unloaded AMP Solutions in Water at Atmospheric Pressure^a^

	η [mPa·s]						
*T* [°C]	*w*_AMP_ = 0.05	*w*_AMP_ = 0.15	*w*_AMP_ = 0.30	*w*_AMP_ = 0.50	*w*_AMP_ = 0.70	*w*_AMP_ = 0.90	*w*_AMP_ = 0.95
20	1.25	2.06	4.85	15.56	43.23		
30	0.97	1.51	3.24	9.35	23.15		
40	0.78	1.16	2.31	5.86	13.39	41.73	48.20
50	0.64	0.92	1.74	3.88	8.36	23.64	25.60
60	0.54	0.75	1.34	2.79	5.60	13.27	14.80
70	0.46	0.63	1.02	2.06	4.86	8.37	9.19
80	0.41	0.53	0.84	1.60	3.47	5.57	6.02

a Standard uncertainties are *u*(*P*) = 0.3 kPa, *u*(*T*) = 0.01 K, *u*_r_(η) = 0.05, and *u*(*w*_AMP_) = 0.0001.

### Experimental Matrix

The experimental matrix for the density and viscosity measurements was defined to cover AMP concentration from 2 to 4 mol/dm^3^, while PZ content was changed from 1 to 1.5 mol/dm^3^. The experimental design is schematized in Table 2, where α is the CO_2_ loading. A symmetrical factorial design is not possible in this case due to the precipitation of PZ at higher concentrations. For example, an aqueous solution of 3 mol/dm^3^ PZ and 1.5 mol/dm^3^ AMP was prepared but precipitated at room temperature. A CO_2_-loaded aqueous AMP-PZ mixture can also precipitate at different AMP/PZ concentrations and CO_2_ loadings, and therefore, measurements were not possible for all the concentrations designed, as shown with the red color in Table 2. Solutions of 3 mol/dm^3^ AMP (26.6 mass %) + 1.5 mol/dm^3^ PZ (12.8 mass %) at 0.8  loading, solutions of 4 mol/dm^3^ AMP (35.5 mass %) + 1.5 mol/dm^3^ PZ (12.9 mass %) at 0.4, 0.6, and 0.8  loadings, and solutions of 4 mol/dm^3^ AMP (35.6 mass %) + 1 mol/dm^3^ PZ (8.6 mass %) at 0.6 and 0.8  loadings precipitated at room temperature, and for these solutions, density and viscosity were not measured. It is important to note that some experiments, at high temperatures and high CO_2_ loading, were not possible, since the gold-coated stainless-steel ball used to measure viscosity was blocked. This may happen due to the stripping of CO_2_ under this operative condition, which creates a bubble that blocks the ball, preventing both the density and viscosity measurements.

## Modeling Approach

### Density Modeling

For unloaded solutions, the excess molar volume is commonly used to correlate the density of the amine aqueous solutions. The density (ρ [kg/m^3^]) is linked to the excess molar volume (*V*^exc^ [m^3^/kmol]) as shown in eq 1. Volumetric properties of binary mixtures (density and excess molar volume) can be used to study the solute–solute and solvent–solute interactions and structural effects arising from differences in molar and free volume between solution components.^17^
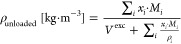
1
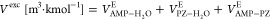
2where *x*_*i*_ is the component mole fraction, *M*_*i*_ [kg/kmol] is the molecular weight, and ρ_*i*_ [kg/m^3^] is the density of the pure component.

A linear correlation as a function of temperature has been developed to describe the density of a pure AMP solution. The correlation has been fitted to the experimental data by Na et al.^18^ and Chan et al.^19^ due to the high purity of AMP used in these works, i.e., >99%. PZ is solid up to 111.4 °C; therefore, in this work, the density of pure piperazine was fixed to its value at the melting point, 1100 kg/m^3^, based on the DIPPR.^20^ The parameters are listed in Table S2.

The excess molar volume is correlated to the Redlich–Kister equation in eq 3. The following assumptions and simplifications have been made:

- Additive effect for the excess molar volume of the binary mixtures.
- Third-order effects are neglected.
- The contribution to the excess volume for AMP-PZ has been obtained by AMP-PZ-H_2_O data.

The last assumption has been considered due to the high precipitation tendency of AMP and PZ, which makes it impossible to measure the physical properties of AMP/PZ solutions when they are in the solid state.

For CO_2_-loaded solutions, a correlation was developed taking as a basis the one proposed by Hartono et al.^13^ (eqs 5 and 6).
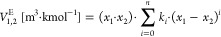
3
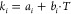
4
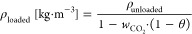
5
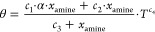
6

In eqs 4 and 6, *a*_*i*_, *b*_*i*_, and *c*_*i*_ are the fitted parameters, *n* defines the order of the polynomial expansion, and *T* is the temperature in Kelvin. In eqs 5 and 6,  is the mass fraction of CO_2_, *x*_amine_ is the mole fraction of amine (AMP + PZ) in the solution, and θ is the parameter associated with the volume expansion caused by the CO_2_ addition. In eq 6, the mole fraction of the amine is the sum of the mole fractions of AMP and PZ. The underlying assumption is that CO_2_ reacts the same with both AMP and PZ, implying iso-loading. This assumption may not hold as PZ has a higher tendency to carbamate and protonate compared to AMP.^21,22^ Consequently, CO_2_ is expected to be predominantly bound to PZ at lower loadings, resulting in this hypothesis failing. However, this simple approach is used because there are not enough published speciation data for loaded aqueous AMP/PZ solutions and because neglecting this will simplify the model.^2^

Table 3 shows the datasets used in the fitting of the density of aqueous AMP and PZ solutions and aqueous CO_2_-loaded and CO_2_-unloaded AMP-PZ solutions.

**Figure 3:**
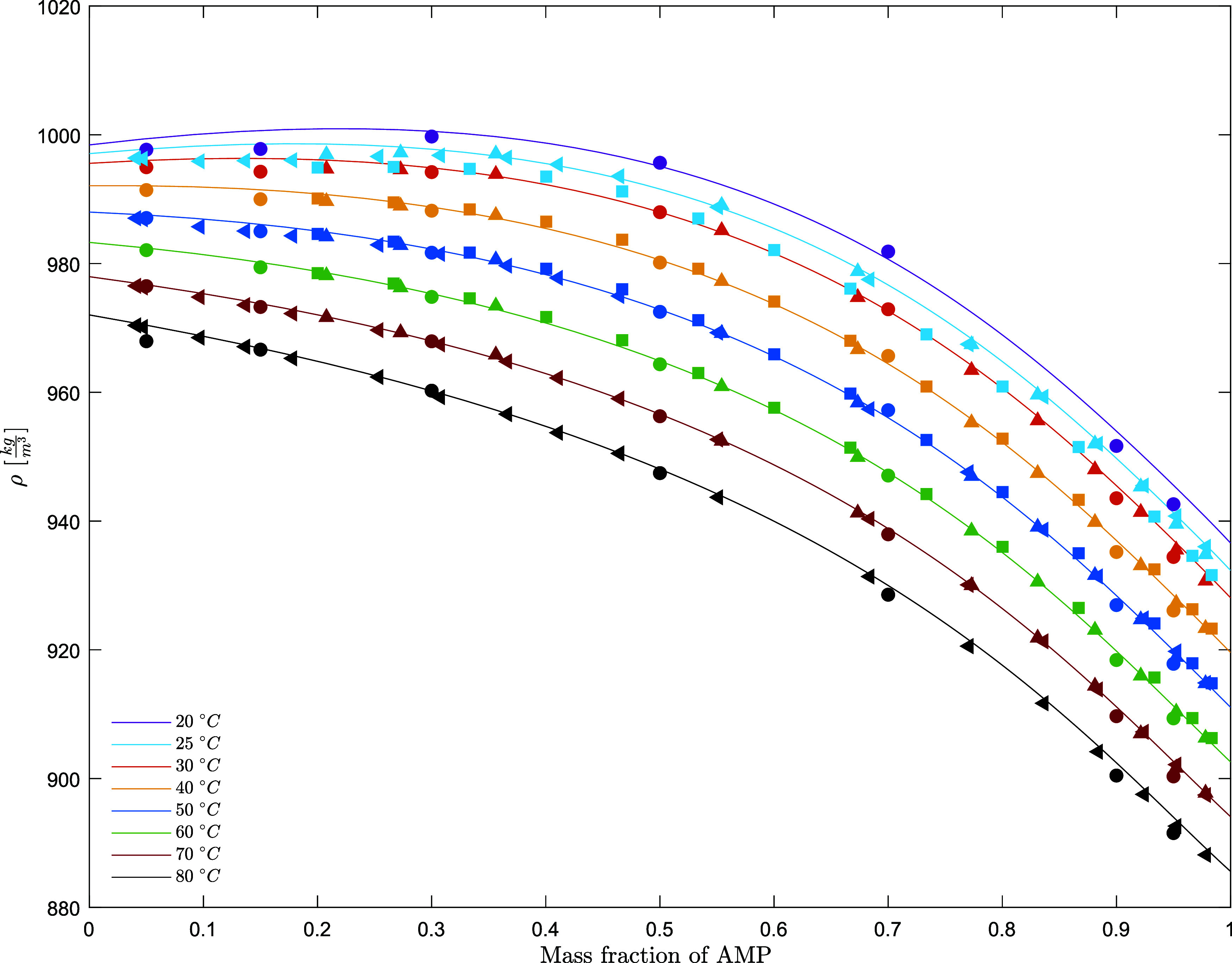
Density of aqueous AMP solutions (points, exp; lines, model) (solid circle, this work; solid up-pointing triangle, Henni et al.;^9^ solid square, Na et al.;^18^ solid left-pointing triangle, Chan et al.^19^).

**Figure 4:**
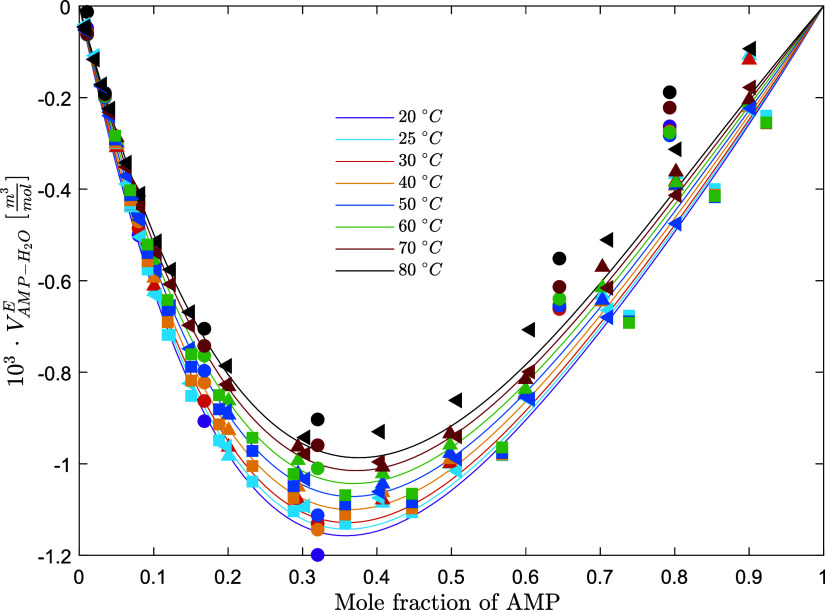
Excess molar volume of aqueous AMP solution (points, exp; lines, model) (solid circle, this work; solid up-pointing triangle, Henni et al.;^9^ solid square, Na et al.;^18^ solid left-pointing triangle, Chan et al.^19^).

### Viscosity Modeling

For unloaded solutions, many different correlations for viscosity have been developed and are available in the open literature and reviewed by Karunarathne.^24^ In analogy to the density modeling, the viscosity deviation, Δη, can be used to investigate the molecular interactions between two or more molecules. The definition used in this work is given by eq 7, where η is the dynamic viscosity [mPa·s], *x*_*i*_ is the mole fraction of the *i* species, and ns is the number of chemical species in the mixture.
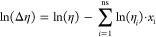
7

The viscosity deviation requires the viscosity of the pure compound. The viscosity of pure AMP has been fitted to the Karunarathne et al.^25^ data to the Andrade equation (eq 8), and the parameters are available in Table S3.

The same approach cannot be used for PZ since it is a solid at a temperature lower than 111.4 °C.^26^ Following the approach used by Pinto and Svendsen,^27^ the viscosity of pure PZ has been set constant and equal to the Eulers number *e*.

This approach is practical but hinders any interpretation of the viscosity deviation. However, it has been employed to ensure consistency between the submodels developed.

In this work, an approach based on a modified Redlich–Kister equation has been used to correlate the viscosity deviation, Δη, to the temperature and amine concentration (eqs 9 and 10).
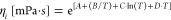
8
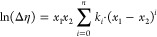
9
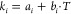
10where *a*_*i*_ and *b*_*i*_ are the fitted parameters, *n* defines the order of the polynomial expansion, and *T* is the temperature in Kelvin.

The viscosity deviation for aqueous AMP + PZ mixtures has been calculated as shown in eq 11.
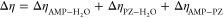
11

The similar assumptions to density modeling have been made:

- Additive effect for the viscosity deviation of the binary mixtures.
- Third-order effects are neglected.
- The contribution to the excess deviation for the blend is obtained by AMP-PZ-H_2_O data.

For CO_2_-loaded solutions, the following correlation has been used (eq 12):
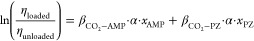
12where α is the CO_2_ loading, *x*_AMP_ and *x*_PZ_ are the mole fractions of AMP and PZ in the solution, respectively, and  and  are the fitted parameters. The hypothesis of iso-loading is assumed for eq 12.

Table 4 shows the datasets used in the fitting of the viscosity of aqueous AMP and PZ solutions and aqueous CO_2_-loaded and CO_2_-unloaded AMP-PZ solutions.

### Optimization Routine and Fitting Procedure

Parameter estimation was performed using an in-house MATLAB routine based on a nonlinear fitting model that minimizes the sum of least-squares errors. To avoid overparameterization of the model, the developed algorithm includes two tests: a parameter significance test (*t* test) and an extra sum of squares test (*F* test). A significance level of α = 0.05 was chosen for these tests. The *t* test and *F* test determine whether the added parameters are statistically significant or provide a statistically significant improvement of the model over an existing set of parameters.

Figure 2 shows a simplified flowchart of the developed algorithm. As illustrated in Figure 2, given a dataset, an initial number of parameters are chosen. To check whether this number is appropriate, the aforementioned tests are performed. For the *F* test, the mean sum of squared errors is calculated for both the full model (MSE_full_), i.e., considering a very high number of parameters, and the partial model (MSE_partial_), a model with a fixed number of parameters for each iteration. The ratio between these two variables is then calculated and compared with the *F* statistic function with ν_full_ and ν_partial_ degrees of freedom. The degrees of freedom are defined in eq 13, where *n* is the number of experimental data points and *p* is the number of parameters used in the fitting.
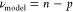
13

The mean sum of square error for the full model (MSE_full_) was calculated in advance, with the full model including a 12^th^-order expansion for the AMP-H_2_O system and an 8^th^-order expansion for the AMP-PZ-H_2_O system.

For the PZ-H_2_O system, a linear expansion model was chosen to avoid extrapolation at higher PZ concentrations, where piperazine is not soluble, as referenced by Pinto and Svendsen^27^ and Muhammad et al.^28^

In the AMP-PZ-H_2_O system, some parameters in the viscosity model are not statistically significant (*p* > 0.05), but they were still included because their removal has been tested to destroy the fitting.

## Results and Discussion

### Density and Viscosity for AMP (1) + H_2_O (3) and PZ (2) + H_2_O (3) Solutions

The measured density and viscosity data for aqueous AMP solutions are listed in Tables 5 and 6. The density and excess molar volume data for the aqueous AMP solutions are depicted in Figures 3 and 4.

**Figure 5:**
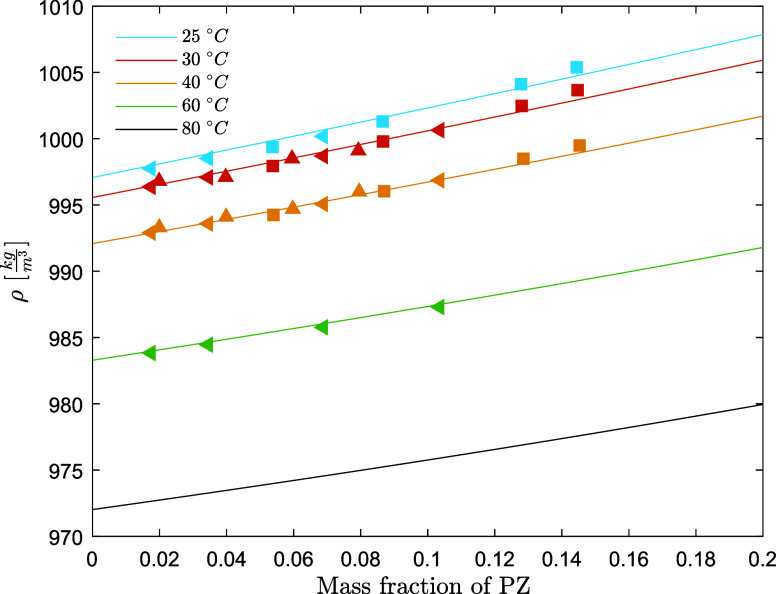
Density of aqueous PZ solutions (points, exp; lines, model) (solid square, Derks et al.;^23^ solid left-pointing triangle, Murshid et al.;^5^ solid up-pointing triangle, Sun et al.^8^).

**Figure 6:**
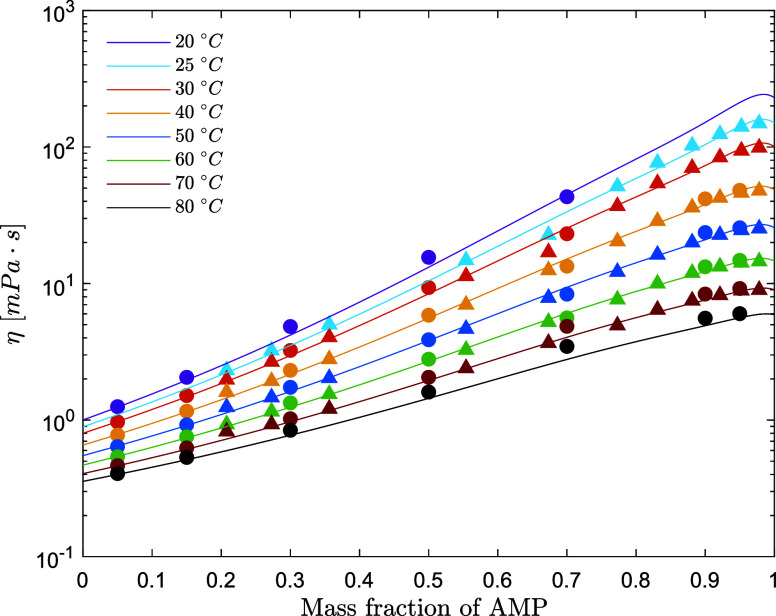
Viscosity of aqueous AMP solutions (points, exp; lines, model) (solid cirle, this work; solid up-pointing triangle, Henni et al.^9^).

**Figure 7:**
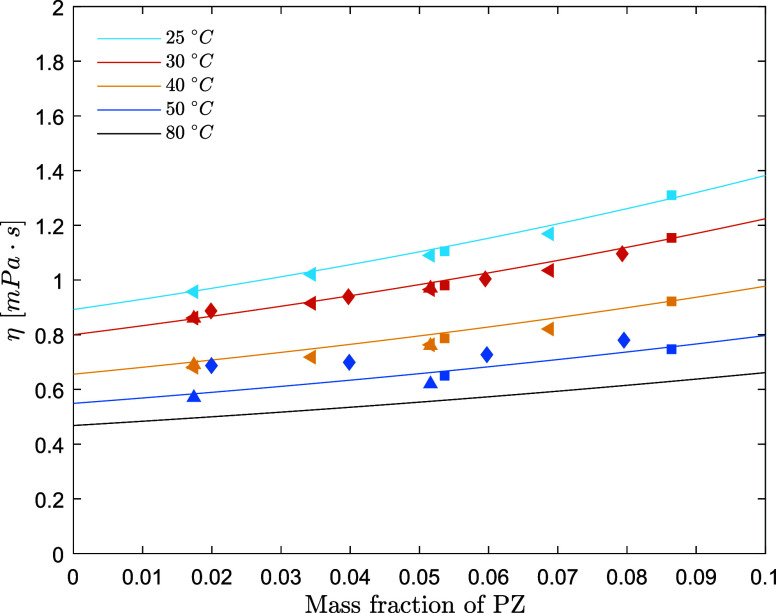
Viscosity of aqueous PZ solutions (points, exp; lines, model) (solid square, Derks et al.;^23^ solid left-pointing triangle, Samanta et al.;^30^ solid diamond, Sun et al.;^8^ solid up-pointing triangle, Muhammad et al.^28^).

**Figure 8:**
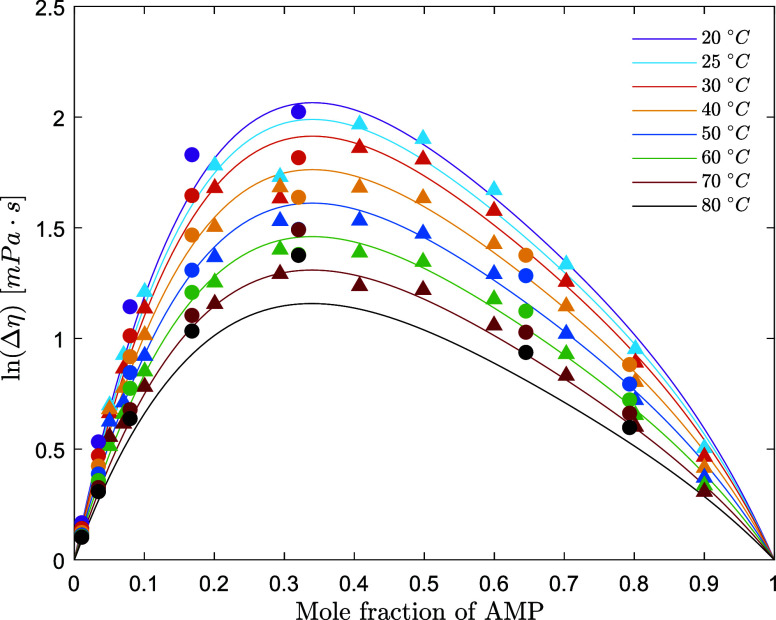
Viscosity excess of aqueous AMP solutions (points, exp; lines, model) (solid circle, this work; solid up-pointing triangle, Henni et al.^9^).

The excess molar volumes for aqueous AMP solutions have been calculated and compared to the experimental data (Figure 4). The experimental data are consistent with each other up to 0.4 mole fraction of AMP, and high scatter in the data is observed at high concentration; this may be due to the purity of AMP used. The excess volume for AMP, , is negative, indicating a volume contraction in the real mixture.^29^ This may be due to the basic nature of AMP and the resulting stronger hydrogen bonding with the H_2_O molecules. The experimental data collected in this work show a crossover in the concentration range of concentration from 5 to 30 mass % at 20 °C, where the density does not decrease monotonically as the concentration of AMP increases as observed with the higher temperatures. This trend was observed also by Henni et al.^9^ and Chan et al.^19^ at 25 °C. This may be due to the high non-ideality of the system at lower temperatures and low concentration of AMP.

The densities of aqueous AMP and aqueous PZ solutions are predicted respectively within 0.06 and 0.02% AARD and within 0.6 and 0.4 kg/m^3^ average absolute deviation (AAD). Figures 3–5 show the performance of the models on the fitted data set for the excess molar volume and density data.

The viscosities of aqueous AMP and aqueous PZ solutions are predicted respectively within 4.0 and 3.6% AARD and within 1.00 and 0.031 mPa·s AAD. Figures 6–8 show the performance of the models on the fitted datasets for the viscosity and viscosity deviation data.

The experimental viscosity data collected in this work align better with the data set collected by Henni et al.^9^ Ghulam et al.^10^ used AMP with certified purity >95%, while Henni et al.^9^ used AMP with certified purity >97%. The data collected in this work and the data by Henni et al. have been used for the parameter fitting. The viscosity deviation has been calculated for the experimental data collected in this work and by Henni et al.^9^ The viscosity deviation for AMP + H_2_O solutions is positive, and this is commonly associated with a negative excess molar volume. This indicates that the viscosity of the real mixture is higher than the viscosity of the ideal solution (where the viscosity excess is equal to zero), and this is due to the volume contraction (negative excess volume) and the resulting more compact intermolecular structure. The viscosity deviation decreases as the temperature increases, suggesting that at high temperatures, where the molecular motion is higher, the system tends to be more ideal than that at lower temperatures. It is important to mention that both the viscosity excess and volume excess become fictitious at 31 °C, which is the melting point of pure AMP.

The Redlich–Kister parameters of the excess molar volume and the viscosity deviations for the binary systems, AMP + H_2_O and PZ + H_2_O, are available in Table 7.

**Table 7:** Redlich–Kister Parameters for the Density and Viscosity of AMP (1) + H_2_O (3) and PZ (2) + H_2_O (3) Solutions

coefficient	unit	value	standard error	*p*
Redlich–Kister Parameters for Density				
		–6.9860 × 10^–3^	4.2962 × 10^–4^	0.0000
	K^–1^	9.3935 × 10^–6^	1.3370 × 10^–6^	0.0000
		4.7047 × 10^–3^	7.9434 × 10^–4^	0.0000
	K^–1^	–7.4078 × 10^–6^	2.4721 × 10^–6^	0.0031
		–4.5580 × 10^–3^	1.6429 × 10^–3^	0.0060
	K^–1^	1.2042 × 10^–5^	5.1107 × 10^–6^	0.0193
		–1.4957 × 10^–2^	1.2036 × 10^–3^	0.0000
		6.2385 × 10^–5^	3.8756 × 10^–6^	0.0000
Redlich–Kister Parameters for Viscosity				
		2.3825 × 10	9.0100 × 10^–1^	0.0000
	K^–1^	–5.5700 × 10^–2^	2.8100 × 10^–3^	0.0000
		–1.1680 × 10	1.8340 × 10	0.0000
	K^–1^	2.5700 × 10^–2^	5.7400 × 10^–3^	0.0000
		1.3672 × 10	3.8330 × 10	0.0005
	K^–1^	–3.3800 × 10^–2^	1.2000 × 10^–2^	0.0057
		5.9596 × 10	6.9500 × 10	0.0000
	K^–1^	–1.3700 × 10^–1^	2.2200 × 10^–2^	0.0000

### Density of AMP (1) + PZ (2) + H_2_O (3) + CO_2_ (4)

**Figure 9:**
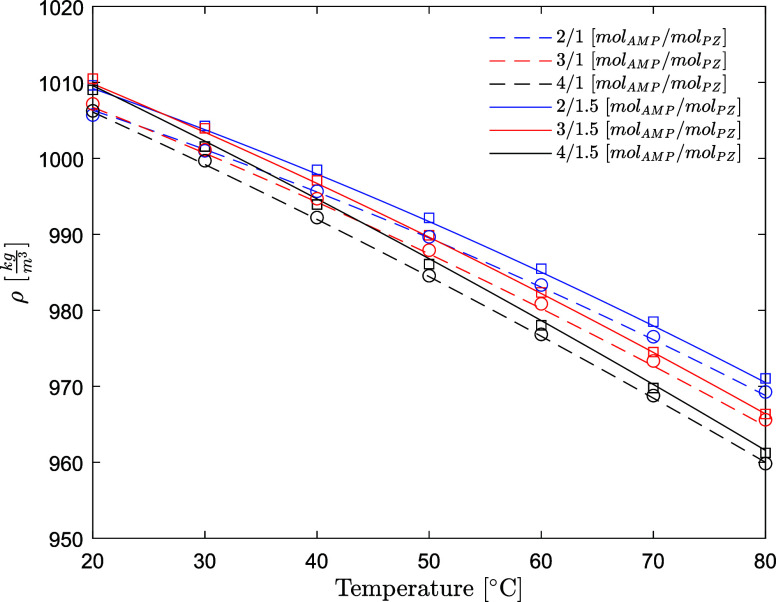
Density of aqueous AMP/PZ solutions without CO_2_ (points, exp; lines, model) (open circle, this work at fixed *w*_PZ_ = 0.086 ([PZ] = 1 mol/dm^3^); open square, this work at *w*_PZ_ = 0.128–0.129 ([PZ] = 1.5 mol/dm^3^)).

The density data for aqueous AMP/PZ/CO_2_ collected in this work are available in Table 8. The density decreases with the temperature and increases with the CO_2_ loading for the whole amine concentration span investigated. The density decreases as the concentration of AMP increases and increases as the concentration of PZ increases, as shown in Figure 9. This is in line with the behavior of the single subsystem, AMP-H_2_O and PZ-H_2_O, shown in Figures 3 and 5. The densities of unloaded aqueous AMP/PZ solutions are predicted within 0.04% AARD and 0.4 kg/m^3^ AAD. The densities of CO_2_-loaded aqueous AMP/PZ solutions are predicted within 0.12% AARD and 1.3 kg/m^3^ AAD. The model developed has been validated on the datasets by Stec,^31^ who measured the density for CO_2_-loaded and CO_2_-unloaded aqueous AMP/PZ mixtures at 15 mass % AMP and 5 mass % PZ and 30 mass % AMP and 10 mass % PZ. The results are reported in Figure S5. The AAD ranges from 1.8 to 2.9 kg/m^3^.

**Table 8:** Density Data ρ [kg/m^3^] for Aqueous AMP/PZ Solution at Atmospheric Pressure as a Function of Temperature, Molar AMP/PZ Ratio, and CO_2_ Loading (α)^a^

2 mol/dm^3^ AMP/1 mol/dm^3^ PZ (*w*_AMP_ = 0.178 and *w*_PZ_ = 0.0860)							2 mol/dm^3^ AMP/1.5 mol/dm^3^ PZ (*w*_AMP_ = 0.177 and *w*_PZ_ = 0.129)						
*T* [°C]	α = 0	α = 0.09	α = 0.18	α = 0.36	α = 0.54	α = 0.82	*T* [°C]	α = 0	α = 0.09	α = 0.19	α = 0.39	α = 0.60	α = 0.80
20	1005.7	1016.5	1027.6	1049.9	1073.6	1091.2	20	1009.6	1022.3	1035.0	1061.1	1087.9	1109.1
30	1001.0	1011.9	1023.2	1045.5	1069.0	1086.7	30	1004.3	1017.2	1030.0	1056.3	1083.0	1104.0
40	995.7	1006.8	1018.2	1040.5	1063.8	1081.6	40	998.5	1011.6	1024.6	1051.1	1077.7	1098.9
50	989.7	1000.8	1012	1034.2	1056.4	1075.5	50	992.2	1005.4	1018.5	1045.2	1071.9	1093.4
60	983.3	994.5	1006.1	1028.6	1050.8	1070.2	60	985.5	998.9	1012.2	1039.2	1065.9	1087.5
70	976.5	987.9	999.3	1022.0	1043.9		70	978.5	992.2	1005.6	1032.8	1059.4	
80	969.3	980.9	992.4	1015.4	1037.5		80	971.0	984.9	998.6	1026.2	1053.0	

3 mol/dm^3^ AMP/1 mol/dm^3^ PZ (*w*_AMP_ = 0.267 and *w*_PZ_ = 0.0860)							3 mol/dm^3^ AMP/1.5 mol/dm^3^ PZ (*w*_AMP_ = 0.266 and *w*_PZ_ = 0.128)						
*T* [°C]	α = 0	α = 0.10	α = 0.21	α = 0.42	α = 0.65	α = 0.86	*T* [°C]	α = 0	α = 0.11	α = 0.20	α = 0.41	α = 0.59	
20	1007.2	1022.5	1036.7	1070.6	1101.3	1116.8	20	1010.5	1030.4	1045.7	1080.8	1110.6	
30	1001.1	1016.6	1025.2	1065.1	1097.4	1118.1	30	1004.0	1024.0	1039.9	1075.2	1104.8	
40	994.7	1010.3	1019.1	1058.9	1090.8	1112.0	40	997.1	1017.5	1033.6	1069.3	1098.7	
50	987.9	1003.8	1012.6	1052.6	1084.3	1105.9	50	989.9	1010.7	1027.1	1063.1	1092.8	
60	980.9	996.9	1005.8	1045.9	1077.2		60	982.4	1003.6	1020.4	1057.0	1085.5	
70	973.3	989.6	998.7	1038.9	1069.4		70	974.5	996.2	1013.3	1050.4	1079.4	
80	965.6	982.2	991.3	1031.7			80	966.4	988.5	1005.8	1043.2	1072.5	

4 mol/dm^3^ AMP/1 mol/dm^3^ PZ (*w*_AMP_ = 0.356 and *w*_PZ_ = 0.0860)							4 mol/dm^3^ AMP/1.5 mol/dm^3^ PZ (*w*_AMP_ = 0.355 and *w*_PZ_ = 0.129)						
*T* [°C]	α = 0	α = 0.10	α = 0.19	α = 0.39			*T* [°C]	α = 0	α = 0.12	α = 0.24			
20	1006.3	1025.1	1044.2	1083.3			20	1009.0	1034.6	1058.6			
30	999.7	1018.8	1038.5	1077.3			30	1001.6	1027.6	1051.9			
40	992.2	1011.6	1031.2	1071.3			40	993.9	1020.4	1045.0			
50	984.6	1004.2	1024.1	1064.3			50	986.1	1013.0	1038.1			
60	976.8	996.8	1016.8	1057.0			60	978.0	1005.3	1030.6			
70	968.8	989.1	1009.5	1049.6			70	969.8	997.5	1023.1			
80	959.8	980.3	1000.6	1040.6			80	961.2	989.5	1015.4			

a Standard uncertainties are *u*(*P*) = 0.3 kPa, *u*(*T*) = 0.01 K, and *u*(ρ) = 0.4 kg/m^3^, and expanded uncertainties are *U*(ρ) = 0.8 kg/m^3^, with a 0.95 level of confidence (*k* ∼ 2), *u*_r_(α) = 0.02, *u*(AMP mass fraction) = 0.0001, and *u*(PZ mass fraction) = 0.00001.

The Redlich–Kister parameter values, their standard deviation, the *p*-values for the AMP/PZ/H_2_O system, and the parameters for the correlation developed for AMP*/*PZ*/*H_2_O/CO_2_ are available in Table 9.

**Table 9:** Fitted RK and CO_2_ Parameters for the Densities of Aqueous AMP/PZ and AMP/PZ/CO_2_ Solutions

coefficient	unit	value	standard error	*p*
*a*_123_		–1.5761 × 10^–1^	1.0744 × 10^–2^	0.0000
*b*_123_	K^–1^	4.4531 × 10^–4^	3.3185 × 10^–5^	0.0000
*c*_1_		–5.5771 × 10^–1^	9.5107 × 10^–2^	0.0000
*c*_2_		2.0128	3.3321 × 10^–1^	0.0000
*c*_3_		1.7421 × 10^–1^	5.8404 × 10^–3^	0.0000
*c*_4_		3.7345 × 10^–1^	2.8324 × 10^–2^	0.0000

**Figure 10:**
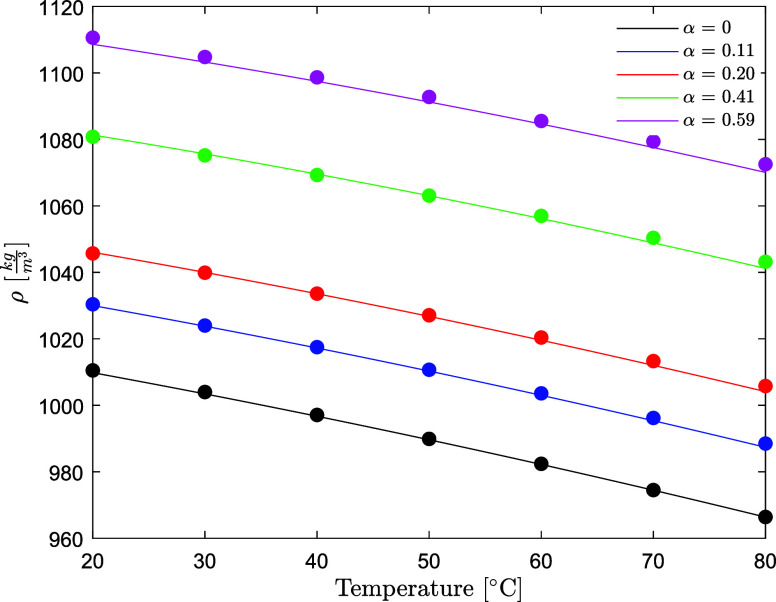
Density of the CESAR1 solvent as a function of temperature and CO_2_ loading (points, this work; lines, model).

The experimental data for the density of the CO_2_-loaded CESAR1 blend are shown in Figure 10.

### Viscosity of AMP (1) + PZ (2) + H_2_O (3) + CO_2_ (4)

The viscosity data for aqueous AMP*/*PZ*/*CO_2_ are available in Table 10.

**Table 10:** Viscosity Data η [mPa·s] for Aqueous AMP/PZ Solution at Atmospheric Pressure as a Function of Temperature, Molar AMP/PZ Ratio, and CO_2_ Loading (α)^a^

2 mol/dm^3^ AMP/1 mol/dm^3^ PZ (*w*_AMP_ = 0.178 and *w*_PZ_ = 0.0860)							2 mol/dm^3^ AMP/1.5 mol/dm^3^ PZ (*w*_AMP_ = 0.177 and *w*_PZ_ = 0.129)						
*T* [°C]	α = 0	α = 0.09	α = 0.18	α = 0.36	α = 0.54	α = 0.82	*T* [°C]	α = 0	α = 0.09	α = 0.19	α = 0.39	α = 0.60	α = 0.80
20	4.17	4.38	4.65	5.22	5.97	6.17	20	5.64	6.01	6.38	7.44	8.80	9.42
30	2.86	3.01	3.23	3.63	4.15	4.37	30	3.71	3.97	4.25	4.98	5.90	6.39
40	2.08	2.20	2.36	2.67	3.03	3.23	40	2.61	2.81	3.02	3.55	4.19	4.61
50	1.54	1.63	1.74	1.97	2.23	2.41	50	1.91	2.06	2.21	2.62	3.09	3.42
60	1.21	1.27	1.37	1.56	1.77	1.92	60	1.46	1.58	1.70	2.02	2.37	2.65
70	0.97	1.02	1.09	1.26	1.42		70	1.16	1.25	1.35	1.60	1.88	
80	0.80	0.85	0.90	1.03	1.17		80	0.94	1.01	1.09	1.31	1.54	

3 mol/dm^3^ AMP/1 mol/dm^3^ PZ (*w*_AMP_ = 0.267 and *w*_PZ_ = 0.0860)							3 mol/dm^3^ AMP/1.5 mol/dm^3^ PZ (*w*_AMP_ = 0.266 and *w*_PZ_ = 0.128)						
*T* [°C]	α = 0	α = 0.10	α = 0.21	α = 0.42	α = 0.65	α = 0.86	*T* [°C]	α = 0	α = 0.11	α = 0.20	α = 0.41	α = 0.59	
20	7.54	8.38	9.05	12.04	15.23	16.11	20	9.87	11.48	12.80	17.44	22.64	
30	4.55	5.09	5.73	7.36	9.36	10.09	30	6.11	7.00	7.98	10.82	13.96	
40	3.11	3.48	3.91	4.96	6.24	6.88	40	4.05	4.63	5.30	7.15	9.15	
50	2.24	2.51	2.82	3.55	4.43	4.97	50	2.87	3.28	3.77	5.07	6.48	
60	1.71	1.91	2.12	2.69	3.34		60	2.14	2.43	2.83	3.79	4.76	
70	1.33	1.49	1.65	2.08	2.57		70	1.64	1.85	2.18	2.92	3.47	
80	1.07	1.19	1.32	1.67			80	1.29	1.45	1.72	2.29	2.75	

4 mol/dm^3^ AMP/1 mol/dm^3^ PZ (*w*_AMP_ = 0.356 and *w*_PZ_ = 0.0860)							4 mol/dm^3^ AMP/1.5 mol/dm^3^ PZ (*w*_AMP_ = 0.355 and *w*_PZ_ = 0.129)						
*T* [°C]	α = 0	α = 0.10	α = 0.19	α = 0.39			*T* [°C]	α = 0	α = 0.12	α = 0.24			
20	11.21	13.30	15.42	22.96			20	17.31	22.36	29.46			
30	6.63	7.84	9.57	13.23			30	9.80	12.63	16.50			
40	4.21	4.98	5.92	8.74			40	6.10	7.82	10.12			
50	2.89	3.40	4.06	5.81			50	4.09	5.21	6.85			
60	2.13	2.50	2.95	4.18			60	2.88	3.61	4.71			
70	1.62	1.89	2.25	3.10			70	2.12	2.70	3.46			
80	1.31	1.46	1.72	2.32			80	1.62	2.07	2.65			

a Standard uncertainties are *u*(*P*) = 0.3 kPa, *u*(*T*) = 0.01 K, *u*_r_(η) = 0.05, *u*_r_(α) = 0.02, *u*(AMP mass fraction) = 0.0001, and *u*(PZ mass fraction) = 0.00001.

**Figure 11:**
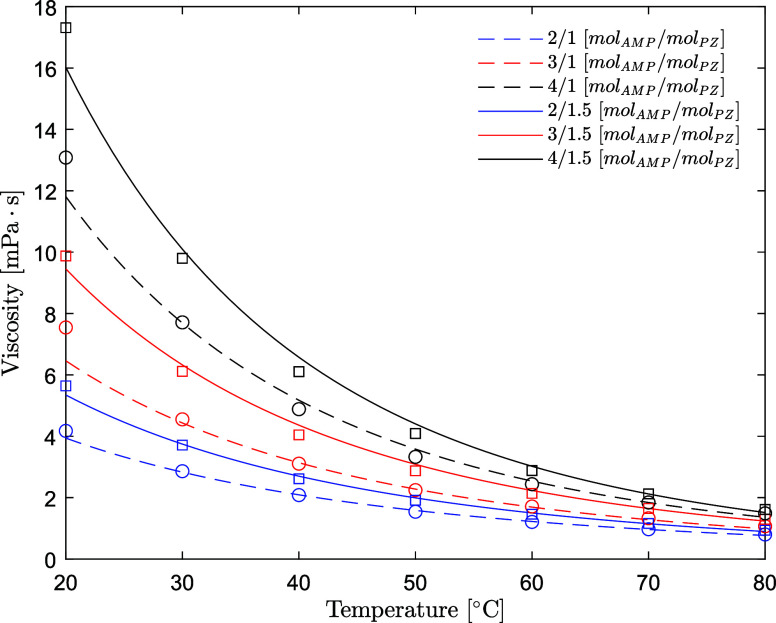
Viscosity of aqueous AMP/PZ solutions without CO_2_ (points, exp; lines, model) (open circle, this work at fixed *w*_PZ_ = 0.086 ([PZ] = 1 mol/dm^3^); open square, this work at *w*_PZ_ = 0.128–129 ([PZ] = 1.5 mol/dm^3^)).

The dynamic viscosity decreases with the temperature and increases with the CO_2_ loading for the whole range of amine concentrations and temperatures investigated. The viscosity increases as the concentration of both AMP and PZ increases. The model predicts the viscosity of unloaded solutions within an AARD of 4.1%, which is considered satisfactory. The model predictions are plotted together with the experimental results in Figure 11. The model tends to systematically underestimate the viscosity at 20 °C, yet this temperature is rarely encountered in an actual plant operation. Viscosity data for aqueous CO_2_-loaded AMP/PZ aqueous solutions have been measured and correlated according to the equation described in Viscosity Modeling. The simple correlation predicts the data within an AARD of 6.1% and an AAD of 0.40 mPa·s over the whole range of amine concentrations and CO_2_ concentrations of this study. Information about the error distribution at the different amine concentrations is available in Table S4. The model developed has been validated on the datasets available in the literature (Fu et al.,^3^ Dash et al.,^4^ Murshid et al.,^5^ Paul and Mandal,^6^ Samanta and Bandyopadhyay,^7^ and Sun et al.^8^). The results are reported in Figure S6; deviation spans from 1 up to 26% AARD*.* The parameters for the CO_2_-unloaded and CO_2_-loaded correlations are available in Table 11.

**Table 11:** Fitted RK and CO_2_ Parameters for the Viscosity of Aqueous AMP/PZ and AMP/PZ/CO_2_ Solutions

coefficient	unit	value	standard error	*p*
		5.414 × 10^2^	3.1868 × 10^2^	0.09823
	K^–1^	–1.630	9.8692 × 10^–1^	0.10748
		–8.634 × 10^3^	1.3410 × 10^4^	0.52389
	K^–1^	4.109 × 10	4.1955 × 10	0.33410
		5.867 × 10^4^	1.2572 × 10^5^	0.64361
	K^–1^	–6.204 × 10^2^	4.2027 × 10^2^	0.14885
		4.453 × 10^4^	1.8197 × 10^4^	0.01957
	K^–1^	3.150 × 10^3^	1.1172 × 10^3^	0.00787
		7.8678 × 10	6.1022	0.0000
		1.4247 × 10^2^	1.4148 × 10	0.0000

**Figure 12:**
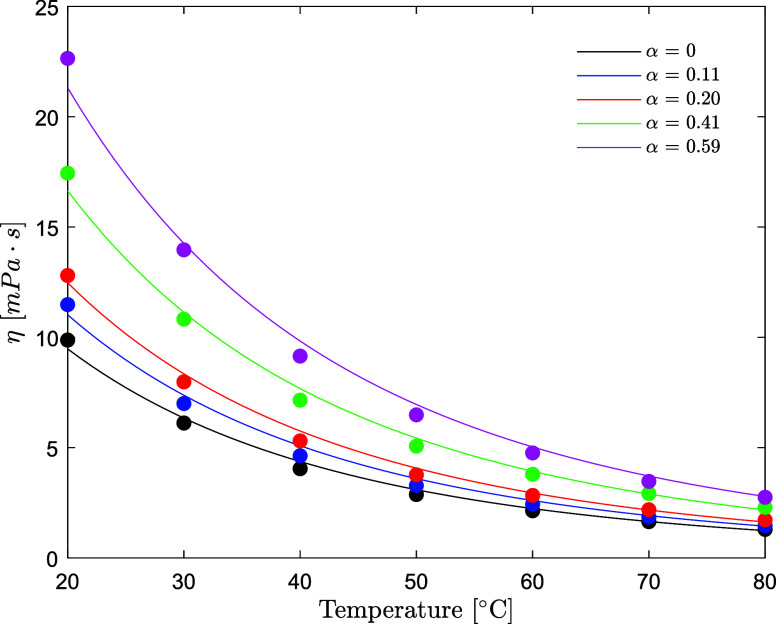
Viscosity of the CESAR1 solvent as a function of temperature and CO_2_ loading (points, this work; lines, model).

The experimental data for the viscosity of CO_2_-unloaded and CO_2_-loaded aqueous AMP/PZ solutions at the CESAR1 concentration and its model prediction are shown in Figure 12. The model predicts the viscosity for the CESAR1 blend within a 5% relative error over the temperature range of 20–80 °C and CO_2_ loading up to 0.59 /mol_amine_.

## Conclusions

In this work, densities and viscosities of CO_2_-unloaded and CO_2_-loaded aqueous AMP/PZ solutions have been measured using a DMA 4500 density meter coupled with a Lovis 2000ME viscosity meter. Physical property data for the CESAR1 blend have been measured in this work.

The densities and viscosities of the aqueous AMP solution have been measured, and the results agree well with the one measured by Henni et al.^9^ Physical property models for aqueous AMP and PZ solutions are proposed in this work.

The viscosity and density models for aqueous AMP solutions account for amine concentrations ranging from 0 to 100 mass % and temperatures up to 80 °C. For aqueous PZ solutions, the density model was fitted using amine concentrations from 0 to 14 mass % and temperatures up to 60 °C, while the viscosity model covers amine concentrations from 0 to 10 mass % and temperatures up to 50 °C. The aqueous PZ solution model behaves logically above these temperatures.

The densities of aqueous AMP solutions and PZ solutions are predicted within 0.02 and 0.06% AARD, respectively.

The viscosities of aqueous AMP solutions and PZ solutions are predicted within 4.0 and 3.6% AARD, respectively.

The models for aqueous AMP/PZ solutions cover amine concentrations of AMP from 2 to 4 mol/dm^3^ and PZ at 1 and 1.5 mol/dm^3^, temperatures up to 80 °C, and CO_2_ concentrations up to 0.86 .

The densities of aqueous AMP/PZ solution increase with the CO_2_ loading, decrease as the concentration of AMP increases, and increase as the concentration of PZ increases. The densities of aqueous AMP/PZ solution are predicted within 0.04 and 0.12% AARD in CO_2_-unloaded and CO_2_-loaded conditions, respectively. The viscosities of aqueous AMP/PZ solution increase with the CO_2_ loading and increase as the concentrations of AMP and PZ increase. The viscosities of aqueous AMP/PZ are predicted within 4.1 and 6.1% AARD in CO_2_-unloaded and CO_2_-loaded conditions, respectively.

The data collected in this work and the correlation developed can be used for modeling the density and viscosity of aqueous AMP/PZ solvents. These models are needed, for example, in the development of thermodynamic and mass transfer models in the CO_2_ capture process. Furthermore, inline density measurements can be used to get information about the CO_2_ loading, making them a valuable means for monitoring a plant operation.

## Supporting Information Available

Density and viscosity data for aqueous 30 mass % MEA (Table S1); correlation parameters for the density of pure AMP and PZ (Table S2); correlation parameters for the viscosity of pure AMP and PZ (Table S3); performance of the viscosity model for CO_2_-loaded AMP/PZ solutions (Table S4); density of aqueous 30 mass % MEA solutions (Figure S1); viscosity of 30 mass % aqueous MEA (Figure S2); absolute relative deviation (ARE) for 30 mass % viscosity measurements compared to the data from this work (Figure S3); model predictions for the density of aqueous CO_2_-loaded AMP/PZ solutions (Figure S4); model predictions for the density of aqueous CO_2_-loaded AMP/PZ solutions (Figure S5); density model predictions for CO_2_-loaded AMP/PZ solution by Stec^31^ (Figure S6); prediction of the viscosity model for AMP/PZ aqueous solutions on literature sources (Fu et al.,^3^ Dash et al.,^4^ Murshid et al.,^5^ Samanta and Bandyopadhyay,^7^ and Sun et al.^8^) (Figure S7) (PDF)
