# Supplementary material for: Density and Viscosity of CO2-Loaded Aqueous 2-Amino-2-methyl-1-propanol (AMP) and Piperazine (PZ) Mixtures
Source: J Chem Eng Data. 2024 Nov 8;70(1):196–207. doi: 10.1021/acs.jced.4c00403 (PMC11726559; doi:10.1021/acs.jced.4c00403)
Supplement: Supplementary file 1 — je4c00403_si_001.pdf [file je4c00403_si_001.pdf]

Supplementary Information

Density and viscosity of CO_2-loaded aqueous 2-amino-2-methyl-1-propanol (AMP) and piperazine (PZ) mixtures

Diego Morlando, Ardi Hartono, Hanna K. Knuutila*

Department of Chemical Engineering, Norwegian University of Science and Technology,

N-7491 Trondheim, Norway.

Corresponding Author: hanna.knuutila@ntnu.no

Uncertainty calculation for the density measurements.

The standard uncertainty for the density measurements has been evaluated using water as reference fluid. The uncertainty has been calculated as:

$$u(\rho) = \sqrt{u^2(\rho_{repeatability}) + u^2(\rho_{calibration})}$$

$$u(\rho_{calibration}) = \sqrt{u^2(\rho_{reference}) + u^2(\rho_{repetability,reference})}$$

Where the first term $u^2(\rho_{repeatability})$, giving information on the repeatability of the measurements, is calculated by the standard deviation of the means of two consecutive measurements. The uncertainty of the apparatus considers the repeatability of the fluid measurements and their accuracy $u(\rho_{reference})$. The term, $u(\rho_{calibration})$, is the standard uncertainties of the measurements ran with water, which was used also as a calibration reference system.

Uncertainty calculation for the mass fraction.

The standard uncertainty for the mass fraction of the amine in the solutions has been estimated as described.

The mass fraction of the component *i* is defined in 1:

$$w_i = \frac{m_i}{m_{tot}} \qquad 1$$

The uncertainty on the mass fraction can be estimated using the law of propagation, 2. The uncertainty on the measured mass $u(m_j)$ has been estimated based on the sensitivity of the scale used to prepare the solutions, $u(m_j) = u(m) = 10^{-6}$ kg, and therefore it is independent of the component.

$$u^2(w_i) = \sum_{j=1}^{N} \left(\frac{\partial w_i}{\partial m_j}\right)^2 \cdot u^2(m) \quad (2)$$

Error Metrics Definition

In this work, the *absolute average error (AAD),* the *absolute average relative error (AARD)*, the *maximum absolute relative error (MARD)* and *the absolute relative error (ARE)* are defined respectively in 3,4, 5, 6.

$$AAD = \sum_{i=1}^{n} \frac{|y_{exp,i} - y_{model,i}\,|}{n} \quad (3)$$

$$AARD = \sum_{i=1}^{n} \frac{|y_{exp,i} - y_{model,i}\,|}{y_{exp,i}} \cdot \frac{1}{n} \quad (4)$$

$$MARD = max\left(\frac{|y_{exp,i} - y_{model,i}\,|}{y_{exp}}\right) \quad (5)$$

$$ARE = \frac{|y_{exp,i} - y_{model,i}\,|}{y_{exp,i}} \cdot 100 \quad (6)$$

Where $y_{exp,i}$ is the experimental property measured, $y_{model,i}$ is the model prediction, n is the number of experiments.

Density and Viscosity Validation Using 30 mass % MEA.

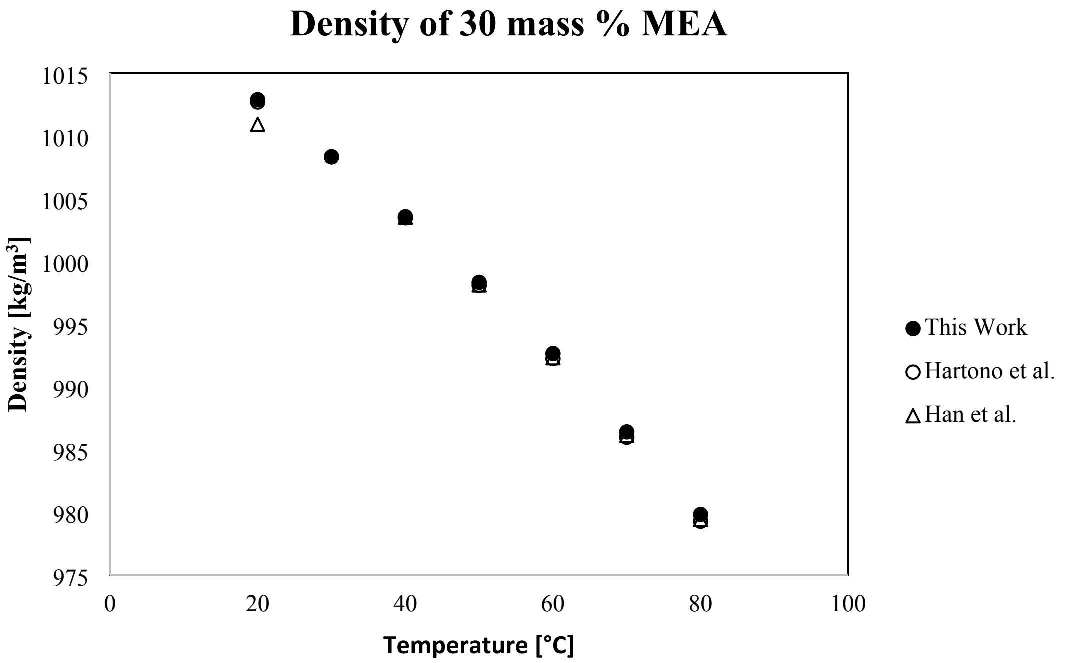

Figure S1 : Density of aqueous 30 mass % MEA *solutions: ● This work, ○ Hartono, et al.* [1], *Δ Han, et al.* [2].

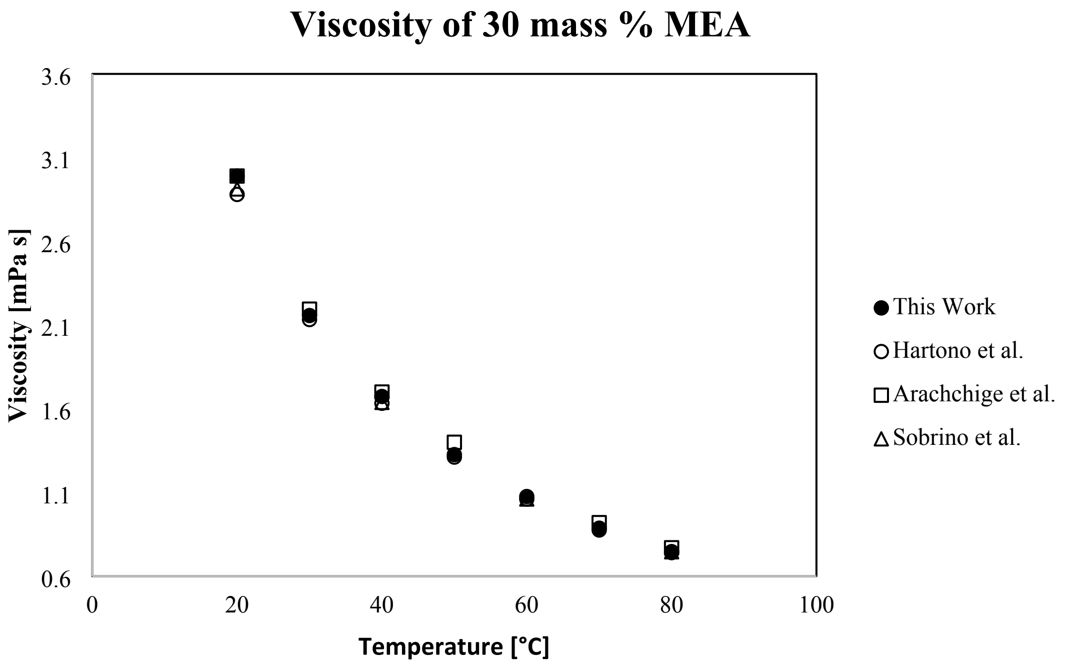

Figure S2 Viscosity of 30 mass % aqueous MEA, ●This work, ○ Hartono, et al. [1], *□ Arachchige* [3], *Δ Sobrino, et al.* [4].

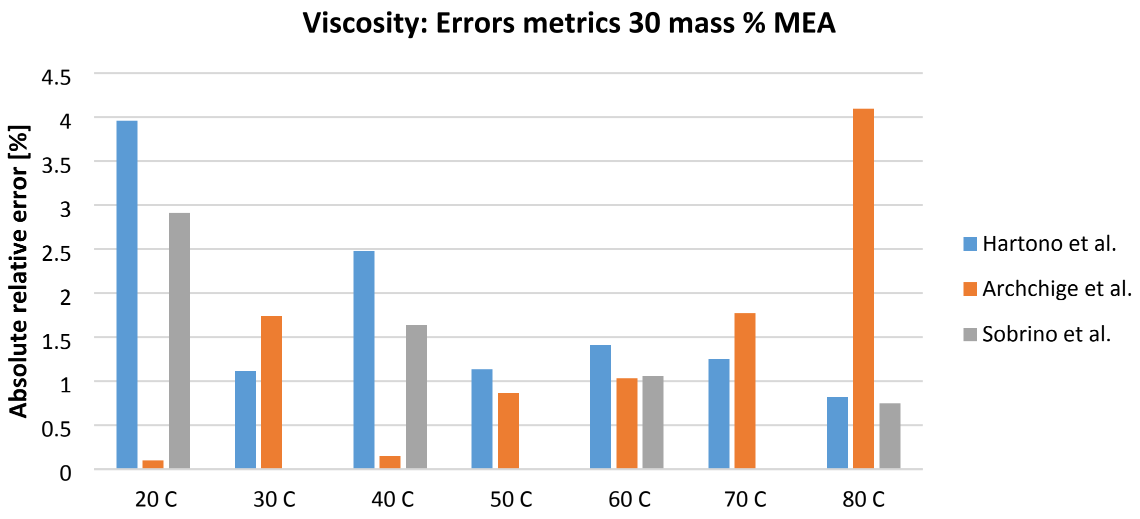

Figure S3: Absolute Relative Deviation (ARE) for 30 mass % viscosity measurements compared to the data from this work. References: Hartono, et al. [1], Arachchige [3], Sobrino, et al. [4].

T [K]	ρ [kg/m^3]	η [mPa s]
293.15	1012.86	2.99
303.15	1008.31	2.16
313.15	1003.54	1.67
323.15	998.31	1.33
333.15	992.64	1.08
343.15	986.39	0.89
353.15	979.81	0.75

Table S1: Experimental Viscosity and Density data for aqueous 30 wt.% MEA at atmospheric pressure. *Standard uncertainties are $u(P)$ = 0.3 kPa, $u(T)$ = 0.01 K, $u(\rho)$ = 0.4 kg/m^3 and expanded uncertainty of $U(\rho)$ = 0.8 kg/m^3, with a 0.95 level of confidence (k ~ 2), $u_r(\eta) =$ 0.05, u(mass fraction MEA) = 0.0001*

Viscosity and Density Modeling

Compound	a_i	b_i
	$Density\ \rho\left[\frac{g}{cm^3}\right] = a_i + b_i \cdot T\ [K]$	
AMP	1.1858	-0.00085
PZ	1.1	-

Table S2: Correlation parameters for the density of the pure component.

Compound	A	B	C	D
		$Viscosity\ \eta\ [mPa \cdot s] = e^{A+\frac{B}{T}+C\cdot\ln(T)+D\cdot T}$		
AMP	-9.51964	12107.48	-8.96225	0.083802
PZ	1	0	0	0

Table S3: Correlation parameters for the viscosity of the pure component.

Density and viscosity model performance of AMP (1) + PZ (2) + H_2O (3) + CO_2 (4)

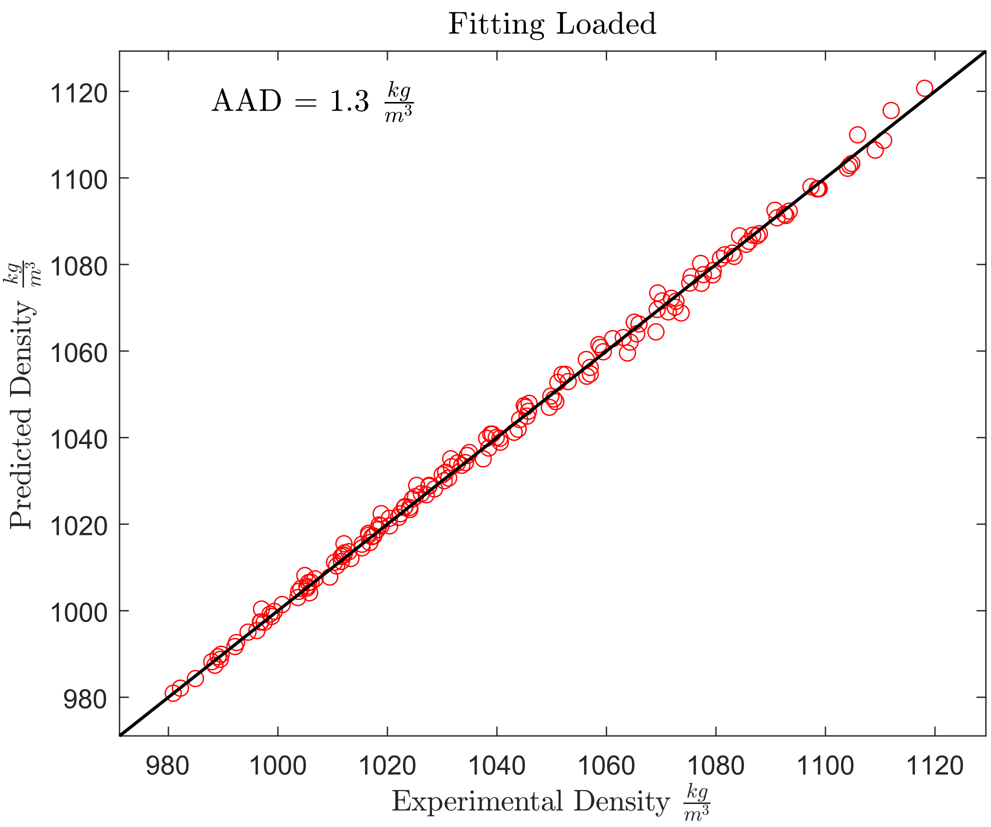

Figure S4: Model predictions of density of aqueous CO_2*-loaded* AMP/PZ *solutions.*

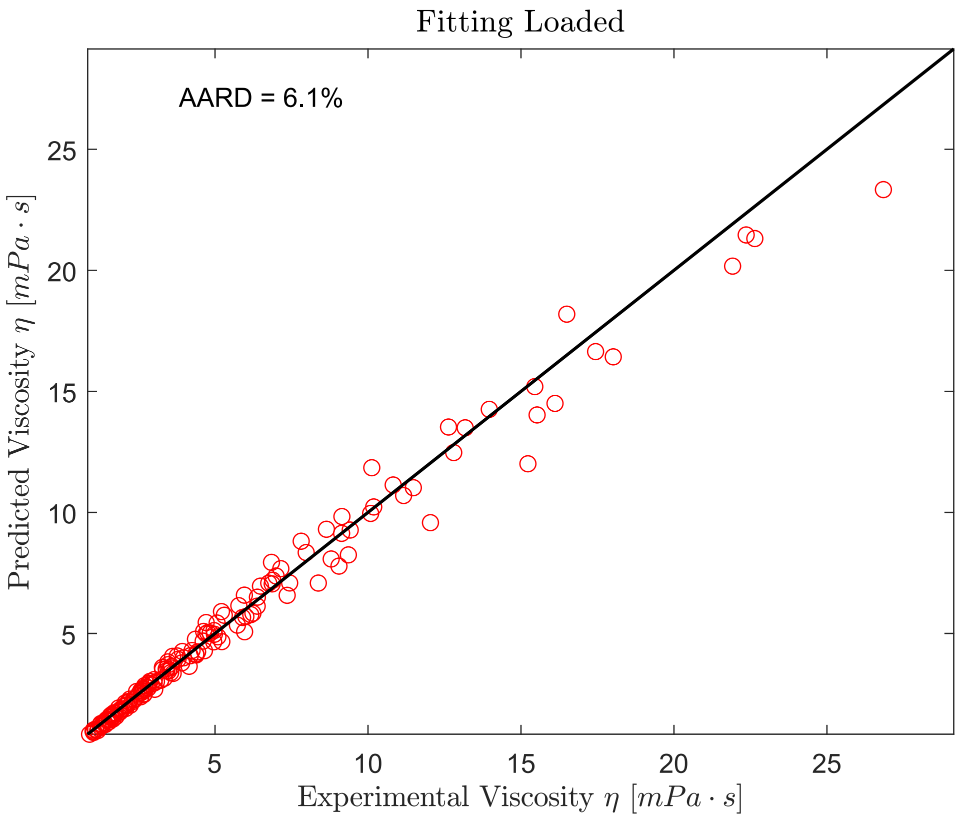

Figure S5: Model predictions of viscosity of aqueous CO_2*-loaded AMP/PZ solutions.*

Table S4: Performance of the viscosity model for CO_2*-loaded AMP/PZ solutions.*

**Amine Concentration**	**CO_2 Loading** $\left[\frac{mol_{CO_2}}{mol_{Amine}}\right]$	**AARD [%]**	**AAD [mPa s]**	**MARD [%]**
2 $\left[\frac{mol}{dm^3}\right]$ AMP 1 $\left[\frac{mol}{dm^3}\right]$ PZ	0-0.82	6.7	0.19	15.2
3 $\left[\frac{mol}{dm^3}\right]$ AMP 1 $\left[\frac{mol}{dm^3}\right]$ PZ	0-0.86	7.4	0.50	21.1
4 $\left[\frac{mol}{dm^3}\right]$ AMP 1 $\left[\frac{mol}{dm^3}\right]$ PZ	0-0.39	4.5	0.50	13.1
2 $\left[\frac{mol}{dm^3}\right]$ AMP 1.5 $\left[\frac{mol}{dm^3}\right]$ PZ	0-0.80	3.4	0.11	11.9
3 $\left[\frac{mol}{dm^3}\right]$ AMP 1.5 $\left[\frac{mol}{dm^3}\right]$ PZ	0-0.59	5.4	0.38	10.5
4 $\left[\frac{mol}{dm^3}\right]$ AMP 1.5 $\left[\frac{mol}{dm^3}\right]$ PZ	0-0.24	9.3	0.74	17.0
**Average**		**6.1**	**0.40**	-

Comparison to the literature: Density Model Prediction.

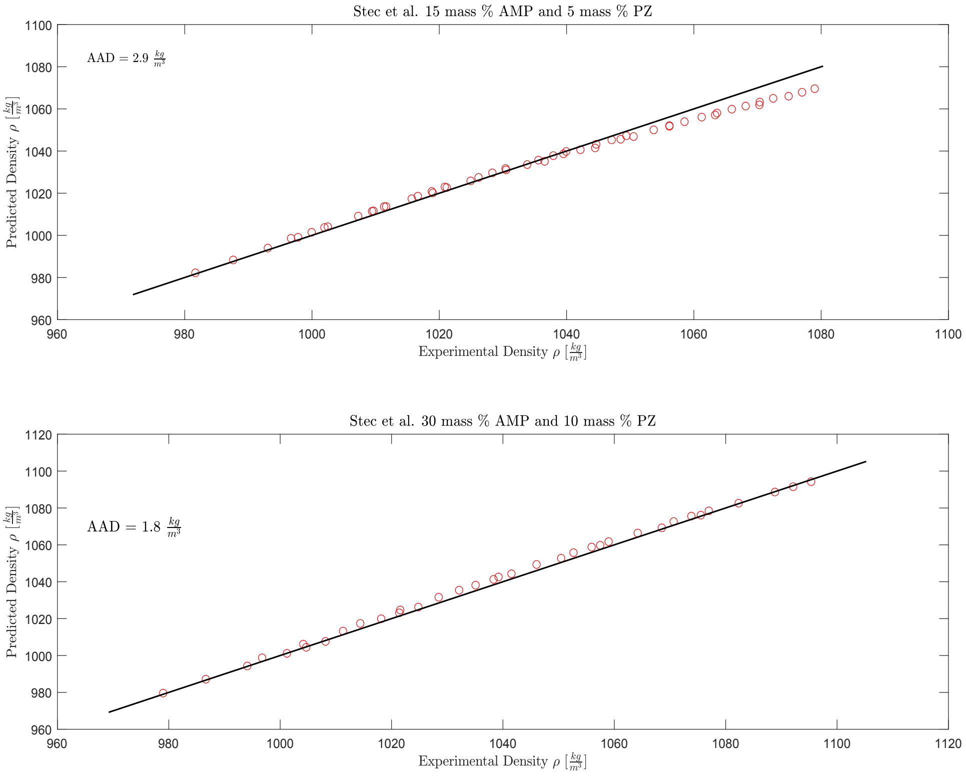

Figure S6: Density Model Prediction for AMP/PZ aqueous, Stec, et al. [5].

Comparison to the literature: Viscosity Model Prediction.

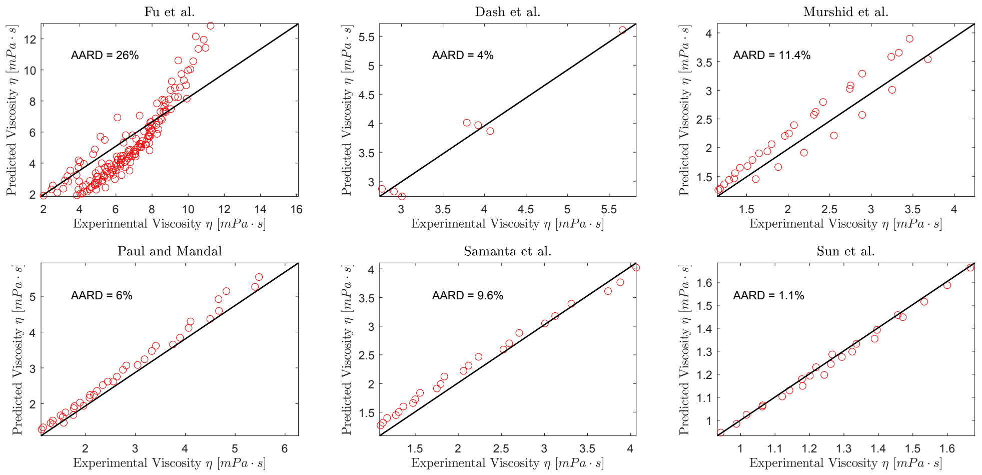

Figure S7: Prediction of the viscosity model for AMP/PZ aqueous solutions on literature sources: Fu, et al. [6], Dash [7], Murshid, et al. [8], Samanta and Bandyopadhyay [9] ,Sun, et al. [10].

References

1 Hartono, A., Mba, E. O. & Svendsen, H. F. Physical Properties of Partially CO2 Loaded Aqueous Monoethanolamine (MEA). *Journal of Chemical & Engineering Data* **59**, 1808-1816 (2014). https://doi.org:10.1021/je401081e

2 Han, J., Jin, J., Eimer, D. A. & Melaaen, M. C. Density of Water (1) + Monoethanolamine (2) + CO2 (3) from (298.15 to 413.15) K and Surface Tension of Water (1) + Monoethanolamine (2) from (303.15 to 333.15) K. *Journal of Chemical & Engineering Data* **57**, 1095-1103 (2012). https://doi.org:10.1021/je2010038

3 Arachchige, U. Viscosities of Pure and Aqueous Solutions of Monoethanolamine (MEA), Diethanolamine (DEA) and N-Methyldiethanolamine (MDEA). (2013).

4 Sobrino, M., Concepción, E. I., Gómez-Hernández, Á., Martín, M. C. & Segovia, J. J. Viscosity and density measurements of aqueous amines at high pressures: MDEA-water and MEA-water mixtures for CO2 capture. *The Journal of Chemical Thermodynamics* **98**, 231-241 (2016). https://doi.org:10.1016/j.jct.2016.03.021

5 Stec, M. *et al.* Density of unloaded and CO2-loaded aqueous solutions of piperazine and 2-amino-2-methyl-1-propanol and their mixtures from 293.15 to 333.15 K. *Physics and Chemistry of Liquids* **54**, 475-486 (2016). https://doi.org:10.1080/00319104.2015.1115328

6 Fu, D., Li, Z. & Liu, F. Experiments and model for the viscosity of carbonated 2-amino-2-methyl-1-propanol and piperazine aqueous solution. *The Journal of Chemical Thermodynamics* **68**, 20-24 (2014). https://doi.org:10.1016/j.jct.2013.08.025

7 Dash, S. K. Absorption of carbon dioxide in piperazine activated concentrated aqueous 2-amino-2-methyl-1-propanol solvent. *Chemical Engineering Science* **66**, 3223-3233 (2011). https://doi.org:10.1016/j.ces.2011.02.028

8 Murshid, G., Shariff, A. M., Keong, L. K. & Bustam, M. A. Physical Properties of Aqueous Solutions of Piperazine and (2-Amino-2-methyl-1-propanol + Piperazine) from (298.15 to 333.15) K. *Journal of Chemical & Engineering Data* **56**, 2660-2663 (2011). https://doi.org:10.1021/je1012586

9 Samanta, A. & Bandyopadhyay, S. S. Density and Viscosity of Aqueous Solutions of Piperazine and (2-Amino-2-methyl-1-propanol + Piperazine) from 298 to 333 K. *Journal of Chemical & Engineering Data* **51**, 467-470 (2006). https://doi.org:10.1021/je050378i

10 Sun, W.-C., Yong, C.-B. & Li, M.-H. Kinetics of the absorption of carbon dioxide into mixed aqueous solutions of 2-amino-2-methyl-l-propanol and piperazine. *Chemical Engineering Science* **60**, 503-516 (2005). https://doi.org:10.1016/j.ces.2004.08.012
